# The Natural History of Metabolic Comorbidities in Turner Syndrome from Childhood to Early Adulthood: Comparison between 45,X Monosomy and Other Karyotypes

**DOI:** 10.3389/fendo.2018.00027

**Published:** 2018-02-09

**Authors:** Yael Lebenthal, Sigal Levy, Efrat Sofrin-Drucker, Nessia Nagelberg, Naomi Weintrob, Shlomit Shalitin, Liat de Vries, Ariel Tenenbaum, Moshe Phillip, Liora Lazar

**Affiliations:** ^1^The Jesse Z and Sara Lea Shafer Institute for Endocrinology and Diabetes, National Center for Childhood Diabetes, Schneider Children’s Medical Center of Israel, Petah Tikva, Israel; ^2^Sackler Faculty of Medicine, Tel Aviv University, Tel Aviv, Israel; ^3^Statistical Education Unit, The Academic College of Tel Aviv Yaffo, Jaffa, Israel; ^4^Pediatric Endocrinology and Diabetes Unit, Dana-Dwek Children’s Hospital, Tel Aviv Sourasky Medical Center, Tel Aviv, Israel

**Keywords:** Turner syndrome, karyotype, metabolic disturbances, obesity, impaired glucose metabolism

## Abstract

**Objective:**

Patients with Turner syndrome (TS) are at increased risk for metabolic disorders. We aimed to delineate the occurrence and evolution of metabolic comorbidities in TS patients and to determine whether these differ in 45,X monosomy and other karyotypes.

**Methods:**

A longitudinal and cross-sectional retrospective cohort study was conducted in a tertiary pediatric endocrine unit during 1980–2016. Ninety-eight TS patients, 30 with 45,X monosomy were followed from childhood to early adulthood. Outcome measures included weight status, blood pressure (BP), glucose metabolism, and lipid profile.

**Results:**

Longitudinal analysis showed a significant change in body mass index (BMI) percentiles over time [*F*(3,115) = 4.8, *P* = 0.003]. Age was associated with evolution of elevated BP [systolic BP: odds ratio (OR) = 0.91, *P* = 0.003; diastolic BP: OR = 0.93, *P* = 0.023], impaired glucose metabolism (HbA1c: OR = 1.08, *P* = 0.029; impaired glucose tolerance: OR = 1.12, *P* = 0.029), and abnormal lipid profile (cholesterol: OR = 1.06, *P* = 0.01; low-density lipoprotein cholesterol: OR = 1.07, *P* = 0.041; high-density lipoprotein cholesterol: OR = 1.07, *P* = 0.033). The occurrence of metabolic comorbidities was similar in 45,X monosomy and other karyotypes. Coexistence of multiple metabolic comorbidities was significantly higher in 45,X monosomy [*F*(1,72) = 4.81, *P* = 0.032]. BMI percentiles were positively correlated with metabolic comorbidities (occurrence and number) in each patient (*r* = 0.35, *P* = 0.002 and *r* = 0.383, *P* = 0.001, respectively).

**Conclusion:**

Our longitudinal study provides unique insights into the evolution of weight gain and metabolic disorders from childhood to early adulthood in TS patients. Since overweight and increasing age aggravate the risk for metabolic comorbidities, careful surveillance is warranted to prevent and control obesity already from childhood. The more prominent clustering of metabolic comorbidities in 45,X monosomy underscores the importance of a more vigorous intervention in this group.

## Introduction

Turner syndrome (TS) is the most common chromosomal abnormality in girls, affecting approximately 1:2,500 of female live births (1). TS results from complete X chromosome monosomy, structural abnormality of the second X chromosome, or mosaicism, and has a characteristic phenotype and various comorbidities (2–4). Young adult women with TS are susceptible to a wide range of medical problems, including autoimmune disorders (5, 6), overweight and obesity (7), an increased risk for metabolic disorders such as glucose intolerance or dyslipidemia (8, 9), and osteopenia/osteoporosis (10). The coexistence of increased weight, impaired glucose metabolism, lipid abnormalities and hypertension ultimately increase their risk for acquired cardiovascular disease (11, 12). While the association between distinctive metabolic derangements and various TS karyotypes has been demonstrated in previous studies (4, 8, 9) the evolution of these comorbidities from childhood to young adulthood in TS patients with various karyotypes has not been thoroughly assessed.

Our institutional policy is to offer continuing surveillance to TS patients till the mid-twenties by a multidisciplinary team aware of the complex and interrelated issues impacting on the health of these individuals. This practice has enabled us to carry out a comprehensive metabolic assessment of TS patients from childhood through puberty to young adulthood and to characterize the natural history of the metabolic comorbidities occurring in young adult women with TS.

The objectives of this longitudinal retrospective study were to assess the occurrence and evolution of overweight/obesity, hypertension, impaired glucose metabolism, and dyslipidemia in TS patients from childhood to early adulthood and to determine whether the prevalence and the natural history of these comorbidities differ in TS patients with 45,X monosomy and with other karyotypes.

## Patients and Methods

### Patients

The medical files of 103 patients with TS confirmed by karyotyping who were followed at the Institution for Pediatric Endocrinology at the Schneider Children’s Medical Center of Israel between the years 1980 and 2016 were reviewed. Ninety-eight cases fulfilled the inclusion criteria: karyotype documentation in the medical files; referral to our clinic prior to pubertal induction; and regular auxologic and blood pressure (BP) surveillance. Five patients [45,X (*n* = 3) and mosaicism 45,X/46,XX (*n* = 2)] were excluded from the study due to severe congenital malformations predisposing to hypertension (severe congenital cardiovascular disease and congenital anomalies of kidneys and urinary tract). The study cohort was categorized by karyotype into two groups: (1) 45,X (monosomy); (2) other karyotypes.

This study was approved by our institutional ethics committee. Because there was no identification of the patients for whom data was retrieved, informed consent by the patients was waived.

### Methods

Our institutional policy consists of continuing clinical and laboratory surveillance of TS patients from referral to the mid-twenties. Girls with TS are routinely scheduled for clinic visits every 4–6 months until attainment of full puberty and adult height, after which they are seen every 6 months until transition to adult endocrine clinics. Clinical assessment includes anthropometric measurements (height, weight), vital signs (heart rate and BP), complete physical examination, and dose adjustment of chronic medications, e.g., growth hormone (GH), estrogen and progesterone, or L-thyroxine. Screening for autoimmune thyroiditis (thyroid function test, antithyroid peroxidase, thyroglobulin antibodies) and celiac disease (anti-tissue transglutaminase, and immunoglobulin A levels) is performed from the age of 4 years onward. Screening for impaired glucose metabolism [fasting plasma glucose, hemoglobin A1c (HbA1c)], dyslipidemia, and liver disease is performed from the age of 8 years onward. All TS patients in whom cardiovascular defects were not identified at diagnosis undergo a reassessment of the cardiovascular system at 5-year intervals.

#### Data Collection through Childhood and Early Adulthood

The data obtained from the medical files included age at diagnosis, age at initiation and cessation of GH therapy, age at onset of spontaneous puberty and/or initiation of estrogen treatment, age at first menstrual bleeding, age at diagnosis of associated disorders (autoimmune diseases, hypertension, impaired glucose metabolism, dyslipidemia), and the use of chronic medications. The clinical and laboratory data (height, weight, pubertal stage, BP, glucose, both fasting and in 2-h postoral glucose tolerance, fasting insulin levels and HbA1c, lipid profile, and liver function tests) were extracted from the medical files at four timepoints: childhood (prior to pubertal induction), adolescence (1–2 years following onset of spontaneous puberty or initiation of estrogen replacement therapy), young adulthood (fully pubertal; age < 21 years) and early adulthood (age > 21 years; prior to transfer to adult endocrine clinics).

#### Body Mass Index (BMI) Assessment

Body mass index (weight in kilograms/square of height in meters) was calculated using the anthropometric measurements documented in the medical files. The evolution of BMI of the TS cohort from childhood through adolescence and young adulthood to early adulthood was assessed by using BMI percentiles. In childhood and adolescence, BMI values were converted to age- and sex-specific percentiles according to the CDC2000 (13). In adulthood, BMI values were converted according to the anthropometric reference data for all ages of the US population in 2003–2006 found in the National Health and Nutrition Examination Survey and National Center for Health Statistics (14). BMI percentiles were used as the index of body weight: underweight, <5th percentile; normal weight, ≥5th to <85th percentiles; overweight, ≥85th to <95th percentiles; and obese, ≥95th percentile (15).

#### BP Assessment

Blood pressure was measured according to the recommendations of the National High Blood Pressure Education Program (NHBPEP) (16). In childhood, percentiles for systolic BP and diastolic BP were calculated according to height, sex, and age (16). Normal BP, prehypertension, and hypertension were defined according to the NHBPEP: BP was defined as normal when BP values were <90th percentile, prehypertension—when either systolic and/or diastolic BP levels were ≥90th to the 95th percentile, hypertension—when either systolic and/or diastolic BP level ≥95th percentile. Hypertension was classified as either stage 1 (≥95th to the <99th percentile plus 5 mm Hg) or stage 2 (≥99th percentile plus 5 mm Hg). In adulthood, BP values were classified as: normal, <120/80; prehypertension, 120–139/80–89; stage 1 hypertension, 140–159/90–99; stage 2 hypertension, ≥160/100 (17).

#### Glucose Metabolism Assessment

Fasting glucose levels were defined as follows: normal, <100 mg/dl; impaired fasting glucose, 100–125 mg/dl; diabetes, ≥126 mg/dl. In those cases which underwent an oral glucose tolerance test (OGTT), 2-h postprandial glucose levels following OGTT were defined as: normal, <140 mg/dl; impaired glucose tolerance, 140–199 mg/dl; diabetes, ≥200 mg/dl. HbA1c levels were categorized as: normal, <5.7%; prediabetes, 5.7–6.4%; diabetes, ≥6.5%. Homeostasis model assessment-insulin resistance (HOMA-IR) was utilized as an insulin resistance index and was calculated by the following equation: [fasting glucose (mg/dl) × fasting insulin (μU/ml)]/405; resistance was defined as HOMA-IR ≥ 3 (18).

#### Lipid Profile Assessment

Total cholesterol (TC), low-density lipoprotein cholesterol (LDL-c), high-density lipoprotein cholesterol (HDL-c), and triglycerides (TGs) were converted to age- and sex-specific percentiles according to the criteria of AAP Lipids in children aged 5–19 years (19). The lipid profile was classified as: desirable <75th percentile/borderline high 75th–90th percentile/high >90th (TC, LDL-c, and TG) and low level <10th percentile/average level 10th–25th/high level >50th percentile (HDL-c). In adults, hypercholesterolemia was defined when TC levels were above 240 mg/dl; elevated LDL-c was defined when LDL-c levels were above 130 mg/dl; hypertriglyceridemia was defined when TG were above 150 mg/dl and low HDL-c was defined as levels <50 mg/dl.

### Statistical Analysis

Data were analyzed using the IBM SPSS software (IBM SPSS Statistics for Windows, Version 24; IBM Corp., Armonk, NY, USA) and the HLM software version 7 (HLM Software). Data are presented as mean and SD or number and percentile, as appropriate. One-way analysis of variance (ANOVA) was used to test for group differences in quantitative, continuous variables; the χ^2^ test (or Fisher’s exact test for small count tables) was used to compare groups in categorical variables. ANOVA with repeated measures was used to test for change in BMI percentile over time and the Friedman test was used to test for change over time in non-quantitative ordinal variables. Growth Curve Models with Logit link functions were used to test odds ratio (OR) of age on the risk of developing various metabolic comorbidities at early adulthood. The Pearson’s correlation coefficient was used to test for association between quantitative variables. Logistic regression was used to test for risk factors for the occurrences of metabolic diseases. A *P*-value of ≤0.05 was considered significant.

## Results

### Clinical Characteristics

The study group consisted of 98 patients with TS, 30.6% (*n* = 30) with 45,X monosomy and 69.4% (*n* = 68) with other karyotypes: mosaicism 45,X/46, XX (*n* = 21), isochromosomes (Xq), or deletions (*n* = 29); a marker or ring chromosome (*n* = 10); karyotypes with Y chromosome material (*n* = 8).

The characteristics of the study population are shown in Table 1. The mean duration of follow-up in this cohort was 11.7 years, with no significant difference between 45,X monosomy and other karyotypes. At diagnosis patients with monosomy were significantly younger than those displaying other karyotypes *(P* = 0.05). Spontaneous puberty occurred less frequently in TS girls with monosomy (*P* = 0.01). Age at onset of spontaneous puberty or age at initiation of pubertal induction were similar in both TS groups, while age at first menstrual bleeding was older in those with monosomy (*P* = 0.006). GH therapy was administered to 56% of the cohort; the rate of GH treatment, the age at initiation of therapy and the duration of therapy were similar in the two groups. Autoimmune thyroiditis was diagnosed in 33% and celiac disease in 8.2% of the cohort, with no significant differences between groups. Liver diseases were found in 22.5% (*n* = 22) of the patients: primary biliary cirrhosis in 2, sclerosing cholangitis in 1, autoimmune hepatitis in 6, fatty liver in 8, and undetermined etiology in 4 patients. In 10 patients, elevated liver enzymes were present already from adolescence.

**Table 1:** Characteristics of Turner syndrome cohort.

	All karyotypes	45,X monosomy	Other karyotypes	*P*
Number of subjects, *n* (%)	98	30 (30.6)	68 (69.4)	
Duration of follow-up (years)	11.7 ± 5.9	11.7 ± 5.8	11.7 ± 6.1	0.996
Age at diagnosis (years)	6.7 ± 4.5	5.4 ± 4.6	7.3 ± 4.4	**0.05**
Spontaneous puberty, *n* (%)	27 (27.6)	3 (10)	24 (35.3)	**0.01**
Age at spontaneous puberty (years)	11.8 ± 1.3	11.5 ± 1.6	11.9 ± 1.3	0.611
Age at pubertal induction^a^ (years)	13.7 ± 1.3	13.8 ± 1.3	13.6 ± 1.4	0.465
Age at first menstrual bleeding^b^ (years)	16.1 ± 1.8	17.0 ± 1.6	15.8 ± 1.8	**0.006**
Growth hormone therapy, *n* (%)	55 (56.1)	20 (66.7)	35 (51.5)	0.162
Age at growth hormone initiation (years)	8.4 ± 2.8	9.1 ± 2.9	8.0 ± 2.7	0.517
Age at last clinic visit (years)	21.3 ± 6.0	21.4 ± 5.8	21.2 ± 6.0	0.900
Autoimmune thyroiditis, *n* (%)	32 (32.7)	13 (43.3)	19 (27.9)	0.193
Celiac disease, *n* (%)	8/94 (8.5)	4/30 (13.3)	4/64 (6.3)	0.262
Liver disease, *n* (%)	22 (22.5)	5 (16.7)	17 (25.0)	0.362

*Data expressed as mean and SD unless otherwise specified*.

*Bold font represents P values which are statistically significant*.

*^a^3 of the TS cohort were <10 years of age at data collection*.

*^b^19 of the TS cohort have not attained menarche; 3 prepubertal and 16 pubertal girls (≤Tanner 4)*.

### Evolution of Weight Status and Metabolic Comorbidities

In childhood, metabolic impairments were detected in 20–25% of the studied girls: overweight/obesity in 15%, elevated systolic and/or diastolic BP in 22%, impaired glucose metabolism in 10%, and increased lipid levels (TG and TC) in 27%.

Longitudinal analysis of weight status (expressed as BMI percentile) at three times points (from childhood to young adulthood) showed a significant change over time in both those with 45,X monosomy and those with other chromosomal abnormalities [*F*(2,132) = 3.1, *P* = 0.05], with a marginally significant difference between groups [*F*(1,66) = 3.7, *P* = 0.06] and with no significant between-group interactions. Longitudinal analysis from childhood to early adulthood showed a significant change in BMI percentiles over time [*F*(3,115) = 4.8, *P* = 0.003]. Sidak *post hoc* analysis showed that BMI percentile at early adulthood was significantly lower than at adolescence or young adulthood.

Longitudinal analysis (using Growth Curve Models) of metabolic comorbidities from childhood to early adulthood showed that age was associated with evolution of elevated BP percentiles (SBP: OR = 0.91, *P* = 0.003; DBP: OR = 0.93, *P* = 0.023), impaired glucose metabolism (HbA1c: OR = 1.08, *P* = 0.029; IGT: OR = 1.12, *P* = 0.029), and abnormal lipid profile (TC: OR = 1.06, *P* = 0.01; LDL-c: OR = 1.07, *P* = 0.041; HDL-c: OR = 1.07, *P* = 0.033).

### Cross-sectional Analysis of Weight Status and Metabolic Comorbidities

#### Weight Status

At childhood and adolescence, the mean BMI percentile of the 45,X monosomy was significantly higher than that of the other chromosomal abnormalities (*P* = 0.037 and *P* = 0.035, respectively), while at young adulthood and early adulthood it was similar in both groups (Tables 2 and 3). The distribution of BMI weight categories (underweight, normal weight, overweight, and obese) at the follow-up timepoints was similar for both groups.

**Table 2:** Cross-sectional analysis of weight status and blood pressure in 45,X monosomy and other karyotypes at childhood, adolescence, young, and early adulthood.

	Childhood			Adolescence			Young adulthood			Early adulthood		
	45,X	Other karyotypes	*P*	45,X	Other karyotypes	*P*	45,X	Other karyotypes	*P*	45,X	Other karyotypes	*P*
Number	27	60		29	62		24	50		18	37	
Age (years)	9.2 (1.0)	9.1 (1.4)	0.777	13.5 (1.3)	13.7 (1.2)	0.455	19.1 (1.2)	19.2 (1.4)	0.852	25.6 (2.3)	25.9 (1.7)	0.514

**Weight status**												
BMI (kg/m^2^)	18 (2.7)	17.0 (2.4)	0.065	21.8 (4.2)	20.3 (3.1)	**0.044**	25.7 (6.1)	23.8 (4.5)	0.132	25.6 (5.1)	25.2 (5.7)	0.784
BMI percentile	65.0 (20.6)	52.5 (27.2)	0.037	68.3 (23.8)	56.1 (26.0)	**0.035**	68.6 (29.1)	61.4 (25.4)	0.279	50.1 (25.3)	48.1 (22.8)	0.763

**Weight categories, %**												
Underweight	0	0	0.173	3.4	1.6	0.117	8.3	2.0	0.151	0	2.7	0.539
Healthy weight	81.5	86.7		65.5	85.5		54.2	78.0		83.3	89.2	
Overweight	7.4	11.6		20.7	9.7		20.8	12.0		16.7	5.4	
Obesity	11.1	1.7		10.3	3.2		16.6	8.0		0	2.7	

**Blood pressure**												
SBP (mmHg)	96.5 (8.2)	95.8 (9.6)	0.761	108.1 (13.5)	105.2 (12.6)	0.319	113.5 (13.3)	110.4 (11.9)	0.318	115.9 (10.9)	112.7 (14.5)	0.420
SBP percentile	57.4 (15.8)	59.9 (17.9)	0.540	62.9 (20.4)	65.0 (21.0)	0.667	63.1 (21.0)	60.6 (19.1)	0.602	NA	NA	

**SBP categories, %**												
Normal SBP	81.5	75	1	65.5	64.5	0.864	70.8	76.0	0.736	72.2	81.1	0.528
Pre-HTN	18.5	18.3		10.3	16.1		12.5	10.0		27.8	13.5	
HTN 1	0	1.7		13.8	9.7		4.2	8.0		0	2.7	
HTN 2	0	3.3		10.3	9.7		12.5	6.0		0	2.7	
DBP (mmHg)	60.4 (6.8)	59.1 (7.5)	0.443	67.3 (10.0)	65.6 (8.2)	0.386	71.5 (8.3)	69.2 (7.9)	0.247	72.0 (10.1)	71.0 (9.2)	0.714
DBP percentile	60.4 (17.9)	59.0 (17.1)	0.731	62.8 (20.4)	64.3 (20.3)	0.717	64.8 (20.8)	63.6 (19.7)	0.809	NA	NA	

**DBP categories (%)**												
Normal DBP	74.1	76.7	1	71.4	66.1	0.282	66.6	68.0	0.432	83.3	86.5	0.837
Pre-HTN	25.9	18.3		7.1	21		16.7	24.0		11.1	8.1	
HTN 1	0	3.3		19.7	9.7		16.7	6.0		5.6	5.4	
HTN 2	0	0		3.6	3.2		0	2.0		0	0	

*Data are presented as mean (SD) or percentage of patients*.

*Bold font represents P values which are statistically significant*.

*BMI, body mass index; SBP, systolic blood pressure; DBP, diastolic blood pressure; HTN, hypertension; NA, not applicable*.

**Table 3:** Cross-sectional analysis of glucose metabolism and lipid profile in 45,X monosomy and other karyotypes at childhood, adolescence, young, and early adulthood.

	Childhood			Adolescence			Young adulthood			Early adulthood		
	45,X monosomy	Other karyotypes	*P*	45,X monosomy	Other karyotypes	*P*	45,X monosomy	Other karyotypes	*P*	45,X monosomy	Other karyotypes	*P*
Age (years)	9.2 (1.0)	9.1 (1.4)	0.777	13.5 (1.3)	13.7 (1.2)	0.455	19.1 (1.2)	19.2 (1.4)	0.852	25.6 (2.3)	25.9 (1.7)	0.514
**Glucose metabolism**
Number	27	60		29	62		24	50		18	37	
FPG (mg/dl)	87.9 (7.1)	82.1 (8.2)	**0.002**	88.2 (9.5)	85.5 (9.5)	0.205	85.4 (9.3)	85.6 (13.3)	0.954	83.1 (9.4)	83.2 (8.4)	0.960
Impaired FPG (%)	0	1.7	1	6.9	6.5	1	8.3	8.0	1	5.6	2.7	0.535
HbA1c (%)	5.3 (0.4)	5.1 (0.3)	0.278	5.2 (0.4)	5.3 (0.4)	0.251	5.3 (1.3)	5.2 (0.3)	0.212	5.3 (0.4)	5.4 (0.4)	0.428

**Oral glucose tolerance test**
Number	7	13		15	33		15	27		8	9	
2 h postload glucose (mg/dl)	117.5 (36.9)	88.4 (14.2)	**0.029**	111.4 (35.5)	119.6 (23.4)	0.352	115.8 (37.8)	125.3 (47.0)	0.25	128.9 (47.7)	132.5 (24.4)	0.838
IGT (%)	14.3	7.7	1	20.0	15.1	0.676	26.7	18.5	0.537	50.0	33.0	0.637
HOMA-IR > 3 (%)	14.3	23.1	1	20.0	21.2	1	33.3	33.3	1	12.5	22.2	1

**Lipid profile**
TC	26	55		27	61		24	44		16	37	
TC (mg/dl)	170 (28.9)	173.6 (26.1)	0.574	180.8 (37.4)	175.8 (32)	0.522	194.4 (36.5)	172.5 (36.1)	**0.020**	197.6 (42.7)	183.1 (33.7)	0.192
≤50th centile (%)	50	43.6	0.555	29.2	45.5	0.436	29.2	45.5	0.436	37.5	35.1	0.496
75th centile (%)	23.1	32.7		16.7	18.2		16.6	18.2		6.3	24.3	
90th centile (%)	15.4	7.3		16.7	15.9		16.6	15.9		12.5	8.1	
≥95th centile (%)	11.5	16.4		37.5	20.5		37.5	20.5		43.8	32.4	
LDL-c, *n*	21	38		22	46		19	37		13	35	
LDL-c (mg/dl)	96.4 (21.8)	101.8 (20.8)	0.360	106.3 (30.6)	102.8 (24.7)	0.616	115.0 (32.2)	100.3 (24.6)	0.063	113.3 (29.7)	108.2 (29.8)	0.599
≤50th centile (%)	61.9	55.3	0.935	36.8	51.4	0.271	36.8	51.4	0.271	38.5	40	0.716
75th centile (%)	28.5	26.3		36.8	35.1		36.8	35.1		15.4	28.6	
90th centile (%)	4.8	7.9		0	5.4		0	5.4		15.4	8.6	
≥95th centile (%)	4.8	10.5		26.3	8.1		26.3	8.1		30.8	17.1	
HDL-c, *n*	21	39		23	47		19	38		13	35	
HDL-c (mg/dl)	56.8 (13.7)	55.4 (11.6)	0.677	56.5 (16.3)	57.4 (11.9)	0.790	56.2 (13.8)	54.2 (15.3)	0.646	63.6 (17.7)	56.7 (12.5)	0.134
≥50th centile (%)	76.2	76.9	0.945	73.7	60.5	0.718	73.7	60.5	0.718	84.6	71.5	0.720
25th centile (%)	14.2	10.3		15.8	39.5		15.8	21.1		7.7	20.0	
10th centile (%)	4.8	7.7		0	18.4		0	7.9		7.7	5.7	
≤5th centile (%)	4.8	5.1		10.5	10.5		10.5	10.5		0	2.9	
TG, *n*	24	43		23	49		20	39		15	35	
TG (mg/dl)	80 (34.8)	84.8 (34.7)	0.587	97.7 (52.3)	85 (33.5)	0.219	104.9 (55.5)	81.9 (25.4)	**0.032**	115.7 (81.3)	91.4 (46)	0.198
≤50th centile (%)	54.2	41.8	0.718	20.0	56.4	**0.039**	40.0	54.4	**0.039**	46.1	40.0	0.434
75th centile (%)	20.8	30.2		15.0	30.8		15.0	30.8		7.7	22.9	
90th centile (%)	8.3	14.0		10.0	5.1		10.0	5.1		7.7	17.1	
≥95th centile (%)	16.7	14.0		35.0	7.7		35.0	7.7		38.5	20.0	

*Data expressed as mean (SD) unless otherwise specified*.

*Bold font represents P values which are statistically significant*.

*n, number; FPG, fasting plasma glucose; IGT, impaired glucose tolerance; HOMA-IR, homeostatic model assessment of insulin resistance; TC, total cholesterol; LDL-c, low-density lipoprotein cholesterol; HDL-c, high-density lipoprotein cholesterol; TG, triglycerides*.

#### Blood Pressure

The distribution of BP categories (normal BP, pre-hypertension, stage 1 and stage 2 hypertension) at the follow-up timepoints was similar for all groups. The prevalence of elevated BP at childhood, adolescence, young adulthood, and early adulthood was 21.8%, 35.2%, 25.7%, and 21.8% for SBP and 23%, 33%, 32.4%, and 14.5% for DBP, respectively.

#### Glucose Metabolism

Fasting glucose, 2-h post-OGTT glucose, and HbA1c levels were within the normal range in most of the studied girls at all follow-up timepoints. Impaired fasting glucose was documented in 6.6% of the girls during adolescence and in 8.1% at young adulthood. Impaired glucose tolerance was documented already from childhood, with increasing prevalence during adolescence, young adulthood and early adulthood (10%, 16.7%, 21.4%, and 41.2%, respectively). Insulin resistance (HOMA-IR) was detected in 20% of the patients tested during childhood and adolescence, and in 33.3% of the patients tested at young adulthood. Glucose metabolism parameters showed no statistical difference between the patients with the 45,X monosomy and those with all other karyotypes.

#### Lipid Profile

At the four follow-up timepoints the mean TC, LDL-c, HDL-c and TG levels of the entire cohort were relatively normal, with no significant difference between the two groups with the exception of higher mean TG levels at young adulthood in the 45,X monosomy group (*P* = 0.032). The distribution of TC and LDL-c percentiles (≤50th%, 75th%, 90th%, and ≥95th%) and of HDL-c percentiles (≥50th%, 25th%, 10th%, and ≤5th%) at the four follow-up timepoints was similar in the two groups; the prevalence of elevated TG categories at adolescence and young adulthood was higher in the 45,X monosomy group (*P* = 0.039 for both timepoints).

### Cooccurrence of Metabolic Comorbidities

Cooccurrence of metabolic comorbidities was found in 55.4% of the cohort. There was a significant difference between those with 45,X monosomy and those with other chromosome abnormalities in both the frequency [79.2% and 44%, χ^2^(1) = 8.1, *P* = 0.004] and number of comorbidities [2.4 ± 1.5 and 1.6 ± 1.3, *F*(1,72) = 4.81, *P* = 0.032].

### Correlations

**Figure 1:**
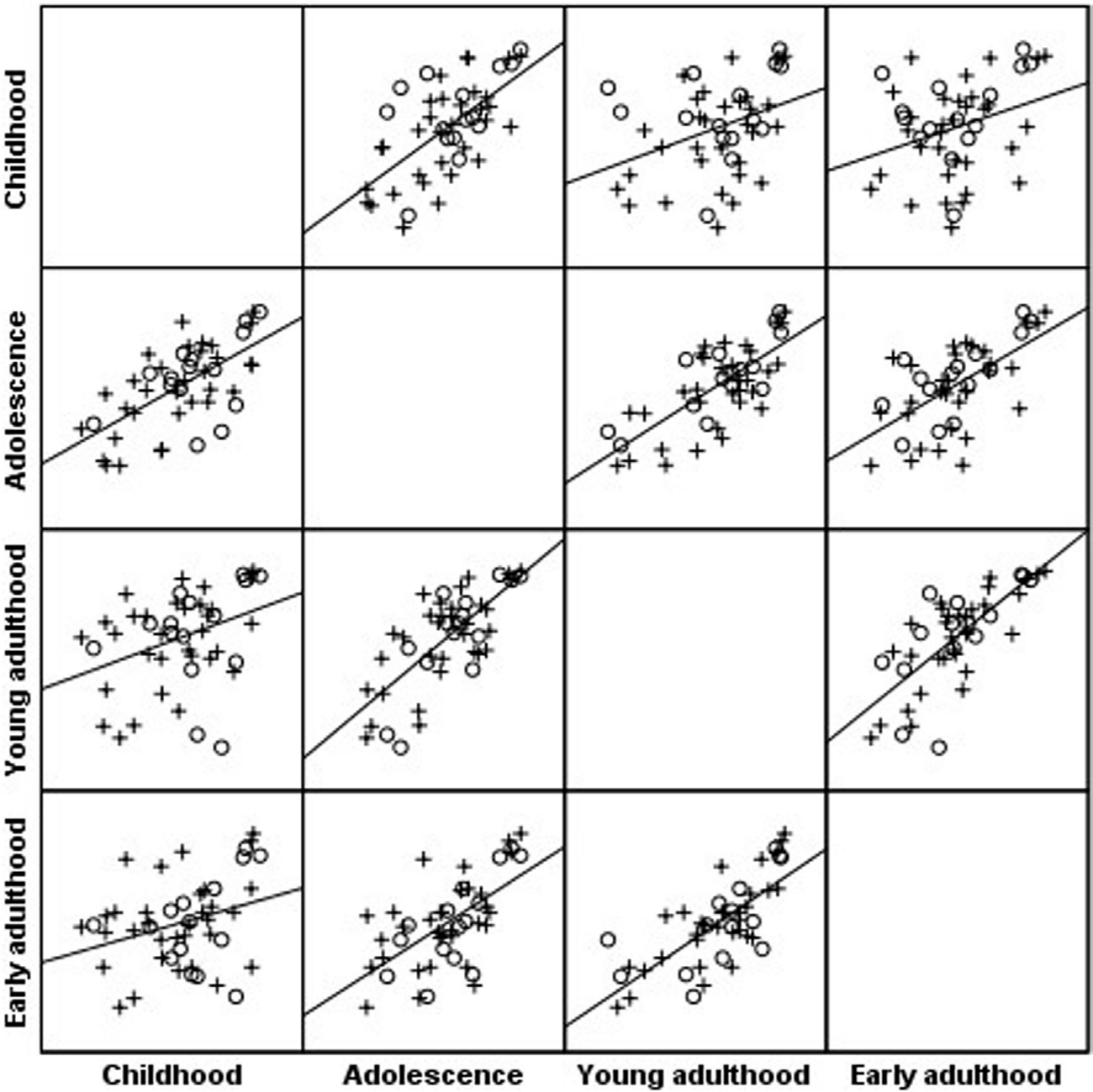
Scatterplot matrix of body mass index (BMI) percentile along four timepoints: BMI percentiles of the Turner syndrome cohort at childhood were positively correlated with BMI percentiles during adolescence (*r* = 0.69; *P* < 0.001), young adulthood (*r* = 0.43; *P* < 0.001), and early adulthood (*r* = 0.33; *P* = 0.021); BMI at adolescence was positively correlated with BMI percentiles during young adulthood (*r* = 0.76; *P* < 0.001) and early adulthood (*r* = 0.65; *P* < 0.001); BMI at young adulthood was positively correlated with BMI percentiles during early adulthood (*r* = 0.76; *P* < 0.001). o, 45,X monosomy, x, all other karyotypes.

Body mass index percentiles of the cohort at childhood were positively correlated with BMI percentiles during adolescence (*r* = 0.69; *P* < 0.001), young adulthood (*r* = 0.43; *P* < 0.001), and early adulthood (*r* = 0.33; *P* = 0.021); BMI at adolescence was positively correlated with BMI percentiles during young adulthood (*r* = 0.76; *P* < 0.001) and early adulthood (*r* = 0.65; *P* < 0.001); BMI at young adulthood was positively correlated with BMI percentiles during early adulthood (*r* = 0.76; *P* < 0.001) (Figure 1).

Body mass index percentiles were positively correlated with the metabolic comorbidities: SBP percentiles (childhood *r* = 0.21, *P* = 0.05; young adulthood *r* = 0.32, *P* = 0.005, early adulthood *r* = 0.35, *P* = 0.009), DBP percentiles (young adulthood *r* = 0.24, *P* = 0.043), FPG (adolescence *r* = 0.24, *P* = 0.020; young adulthood *r* = 0.31, *P* = 0.007; early adulthood *r* = 0.33, *P* = 0.016), HbA1c (early adulthood *r* = 0.38, *P* = 0.010), and TG levels (childhood *r* = 0.27, *P* = 0.028; young adulthood *r* = 0.25, *P* = 0.053). The occurrence of coexistent metabolic comorbidities and the number of comorbidities in each patient were positively correlated with BMI percentile (*r* = 0.35, *P* = 0.002 and *r* = 0.383, *P* = 0.001, respectively).

Logistic regression analysis showed that karyotype, GH therapy, HRT, autoimmune thyroid disease, or celiac disease were not associated with the occurrence of metabolic comorbidities.

## Discussion

Although the cardinal manifestations of TS during childhood and adolescence are short stature and sexual infantilism, a wide spectrum of cardiometabolic risk factors (overweight, hypertension, impaired glucose metabolism and dyslipidemia) also has its start in childhood, increasing the risk for atherosclerosis and cardiovascular disease across the patient’s lifespan (11, 12, 20–23). The evolution of these metabolic disorders from childhood to adulthood has not yet been completely defined. In this longitudinal retrospective study of a relatively large cohort of TS patients followed in our institution from childhood to early adulthood, we found that the prevalence of overweight/obesity and cardiometabolic disorders identified in childhood increased with age. Since adolescence the increased rate and cooccurrence of the metabolic disorders in each patient were associated with overweight/obesity. A more prominent clustering of metabolic comorbidities in 45,X monosomy were found. Our findings confirm previous observations that an abnormal metabolic profile appears at a relatively young age in girls with TS (8, 11, 12, 24–27).

Although the development of the comorbidities from childhood through adolescence to young/early adulthood was similar in all these TS patients, both those with 45,X monosomy and those with other karyotypes, clustering of metabolic risk factors was more prominent in those with 45,X monosomy. It has been suggested that X chromosome gene dosage (complete absence of the second X chromosome or haploinsufficiency of genes on the X chromosome) has an impact on the occurrence of metabolic disorders (9, 28, 29). Our data, however, showed a similar prevalence of overweight, elevated BP, impaired glucose metabolism, and abnormal lipid profile in TS patients with 45,X monosomy and those with other karyotypes, not only during childhood but also through adolescence and young adulthood. These findings are consistent with those of Irzyniec and Jeż, who demonstrated a similar rate of metabolic risk factors in all the karyotype subgroups (30), but in contrast to previous studies reporting a higher rate of metabolic disorders among patients with 45,X monosomy (4, 9, 28, 29). The parental origin of the single X-chromosome has also been linked to the incidence of metabolic disorders. Women with a single maternal X-chromosome were found to be more prone to excessive visceral adiposity and dyslipidemia (9, 31). Regrettably, such an analysis was not performed in our studied cohort and therefore we cannot confirm or negate possible X-chromosome gene imprinting.

A plausible explanation for the early development of metabolic impairments could have been overweight and obesity. However, in a substantial number of the girls in our study already displaying cardiometabolic risk factors during childhood the BMI percentile was normal. As in other studies, our findings showed that hypertension, impaired glucose metabolism and dyslipidemia were not associated with body weight in young girls with TS (7, 24–27). It would therefore appear that these early metabolic derangements may be attributed to risk factors inherent to TS: impaired glucose metabolism—to pancreatic beta cell dysfunction and decreased insulin secretory response to glucose (8, 12); abnormal circadian BP rhythm and elevated BP—to autonomic dysfunction with altered vascular tone (7, 32). It was, however, clear that increased weight aggravated the metabolic profile of these patients in adolescence and young adulthood.

The evolution of weight gain observed in our TS patients was characterized by an increasing rate of overweight and obesity from childhood to young adulthood in the entire cohort, with BMI percentile consistently higher in girls with 45,X monosomy. It is noteworthy that a high BMI percentile in childhood predicted overweight and obesity in adolescence and young/early adulthood. These findings agree with those of earlier and more recent studies (33–36). The natural history of the metabolic risk factors in our study cohort revealed a sustained increase in the prevalence of the metabolic comorbidities from childhood to young/early adulthood. In agreement with previous studies, we found that the occurrence of hypertension, impaired glucose metabolism and hypertriglyceridemia were positively correlated with the increased BMI (7, 12). Interestingly, clustering of several metabolic risk factors was more prevalent in those of the TS patients with 45,X monosomy who displayed increased BMI since adolescence.

In this observational longitudinal study, the issue of causality of the metabolic comorbidities could not be addressed. There is, however, an indication that increased weight during adolescence and young adulthood aggravated insulin resistance, glucose intolerance and hypertriglyceridemia, and contributed to the development of hypertension. Importantly, and similarly to previous reports, we found no evidence that GH treatment, age at onset of spontaneous puberty or at induction of puberty, or estrogen treatment (oral or transdermal) had any impact either on weight gain or on the development of metabolic comorbidities in our patients with TS (37–39).

Obesity, hypertension, impaired glucose metabolism and hyperlipidemia when identified in childhood are all modifiable risk factors. Thus, our findings come to emphasize the importance of regular assessment of weight status and metabolic risk factors in girls with TS from early childhood. Early detection of increased weight gain and of metabolic risk factors allows timely intervention to prevent the overweight and the development of metabolic impairments and their progression toward overt metabolic comorbidities.

The main strength of our study was the longitudinal data obtained from a relatively large cohort of TS patients followed in our tertiary center from childhood to early adulthood. The main limitation of our study is the lack of clinical measures of body adiposity such as skinfold thickness, waist circumference, bioelectrical impedance, or dual-energy X-ray absorptiometry as well as the lack of quantitatively determined atherosclerosis (intimal–medial thickness) by carotid arterial ultrasound. Despite several limitations, BMI is considered a reliable and clinically valid screening tool for obesity. Furthermore, our data include markers of impaired glucose metabolism associated with adiposity such as measures of insulin resistance (HOMA-IR) and IGT. Another limitation is lack of information regarding the origin of the missed X-chromosome.

In conclusion, TS patients followed in our tertiary center from childhood to early adulthood showed a sustained increase in the prevalence of their metabolic comorbidities. While the abnormal metabolic profile during childhood most likely stemmed from risk factors inherent to TS, the aggravation of the metabolic derangements was associated with the increased weight observed during adolescence and young adulthood. Therefore, regular screening of weight and metabolic risk factors and efforts to prevent and control obesity in young TS patients should be accorded a high priority already from childhood. The more prominent clustering of metabolic comorbidities in TS girls with 45,X monosomy and those with overweight/obesity underscores the importance of a still more vigorous intervention in these groups. Future research should address whether modification of these variables at a young age can alter the metabolic outcomes in adulthood.

## Ethics Statement

This study was carried out in accordance with the recommendations of the Rabin Medical Center Institutional Review Board. The protocol was approved by the institutional review board, because there was no identification of the patients for whom data was retrieved, informed consent by the patients was waived.

## Author Contributions

YL and LL contributed to the conception and design of the study, acquisition of the data and interpretation of data, drafted the article, revised it, and gave their final approval of the version to be published. SL contributed to the data analysis, data interpretation, and gave final approval of the version to be published. ES-D contributed to the conception of the study, acquisition of data, and gave her final approval of the version to be published. NN contributed to acquisition of data and gave final approval of the version to be published. NW, SS, LV, AT, and MP contributed to acquisition of data, revised the manuscript critically for important intellectual content, and gave their final approval of the version to be published.

## Conflict of Interest Statement

All authors declare that there is no conflict of interest that could be perceived as prejudicing the impartiality of the research reported.
